# Thyroglobulin levels among iodine deficient pregnant women living in Northern Ireland

**DOI:** 10.1038/s41430-022-01144-z

**Published:** 2022-05-05

**Authors:** Karen Mullan, Paul McMullan, Lucy Kayes, David McCance, Alyson Hunter, Jayne V. Woodside

**Affiliations:** 1 Royal Victoria Hospital (Regional Centre for Endocrinology and Diabetes), Belfast, Antrim UK; 2 Royal Jubilee Maternity Hospital, Royal Victoria Hospital, Belfast, Antrim UK; 3 Centre for Public Health, Institute of Clinical Science, Queens University Belfast, Belfast, UK

**Keywords:** Nutrition, Endocrinology, Biomarkers, Biomarkers

## Abstract

**Background:**

Iodine deficiency has re-emerged among pregnant cohorts in the UK. Thyroglobulin (Tg) is a protein produced uniquely by the thyroid gland which appears to mount a U-shaped response to extremes of iodine status. Tg has been suggested as an alternative marker for chronic iodine deficiency but the value of Tg in pregnancy has not been fully elucidated. A recent non-European study suggested a median Tg ≤10 µg/L with <3% of values >44 µg/L was indicative of sufficiency in the second trimester of pregnancy.

**Methods:**

We measured serum Tg levels in each trimester in 241 pregnant women living in Northern Ireland, a population with mild iodine deficiency at all stages of pregnancy as defined by urinary iodine concentration (UIC) and iodine: creatinine ratio (ICR). Women with Tg antibodies (6% in 1st trimester) were excluded.

**Results:**

The median UIC in this cohort was in the deficient range at 73, 94 and 117 µg/L in sequential trimesters (adequacy ≥ 150 µg/L). Corresponding median Tg levels were 19, 16 and 16 µg/L respectively. Median Tg for all samples was 17 μg/L (IQR 11–31) suggestive of iodine deficiency. Tg was >44 μg/L in 14.3%, 9.4% and 12.4% of women in sequential trimesters respectively. Women with either UIC/ICR below the cut-offs 150 µg/L and 150 µg/g creatinine had higher Tg concentrations in 1st and 2nd trimester (*p* < 0.01; *p* < 0.001) but not in 3rd trimester.

**Conclusion:**

This study adds to the evolving evidence that Tg measurement is of value in reflecting iodine status in pregnancy.

## Introduction

For many years the population of Great Britain and Northern Ireland was thought to have adequate iodine nutrition [1]. The eradication of iodine deficiency from the 1940s onwards was achieved through increased iodine content of dairy products by the addition of iodine to cattle feeds (which was thought to enhance fertility) and the use of iodophors in the milking process. The resulting increase in iodine in the food chain has been described as an ‘accidental public health triumph’ [2].

A re-emergence of mild deficiency across the United Kingdom (UK) and Republic of Ireland (ROI) has been suggested by some but not all recent surveys [3–6]. One survey, in 2011, of >700 UK teenage schoolgirls found the median urinary iodine concentration to be below the level of sufficiency. Levels varied across the nine centres sampled, with the lowest levels found in Northern Ireland (NI) [5]. Different age groups may be more prone to deficiency than others. For example, a survey of primary school age children in the west of NI showed iodine sufficiency [7]. Potential causes for the re-emergence of iodine deficiency include changing farming techniques and dietary habits around dairy consumption in at least some age groups [8].

During pregnancy there is an increase in iodine requirements to compensate for increased urinary losses associated with the increased glomerular filtration of pregnancy, and increased production of maternal thyroid hormones and thirdly placental transfer of iodine for foetal thyroid hormone production [9].

The World Health Organisation (WHO) currently recommends an intake of 250 μg of iodine per day during pregnancy, compared to an intake of 150 μg per day in the adult population, to meet these increasing demands [10].

Severe iodine deficiency during pregnancy is associated with poor cognitive and motor outcomes in the offspring. Moderate deficiency may result in goitre development in the offspring and has been associated with a lowering of intelligence quotient (IQ) by 10–15 points [11, 12]. The effect of mild iodine deficiency during pregnancy is much less clear. Initial findings from a sub-set of the Avon Longitudinal Study of Parents and Children (ALSPAC) (*n* = 1000 and a median UIC 91 µg/L in the 1st trimester) suggested a link between mild maternal iodine deficiency and offspring IQ (age 8), reading ability (age 9) and Key Stage 2 scores (age 11) in a dose dependent fashion [4].

Epidemiological studies assessing iodine nutrition status during pregnancy in the UK are limited to date. To our knowledge there are six geographical areas where iodine nutrition during pregnancy has been studied: North East [13], South East [14], and South West England [4, 15], Oxford [16], Cardiff [17], one study in Scotland [18] and one in NI [19].

Iodine rich food sources include fish and dairy products with the latter found to be the main dietary source of iodine in the UK [20]. With no formal iodine food fortification programme in the UK or ROI, good dietary sources are essential to meet requirements. Food content varies with season and soil content with coastal areas having higher levels [21]. Dietary habits may change during pregnancy, but it is not known how this affects iodine nutrition. Iodine supplementation is currently not recommended during pregnancy in the UK antenatal care guidelines issued by the National Institute of Clinical Excellence (NICE) [22], unlike other nations such as Australia and United States of America (USA). The European Thyroid Association (ETA) guideline on subclinical hypothyroidism includes iodine deficiency as a cause of subclinical hypothyroidism endorsing the WHO intake of 250 µg a day during pregnancy and suggest this should be achieved by administering a supplement containing 150–250 µg of potassium iodide [23].

The WHO currently recommends the use of urinary iodine concentration (UIC) as the gold standard marker of recent dietary iodine intake [24]. Due to large variability, calculation of median population levels from spot urine samples are currently recommended. The WHO defines adequate iodine status during pregnancy as a median population UIC greater or equal to 150 μg/L, which is an increase from the non-pregnancy cut-off value of 100 μg/L [24]. Corrections using urinary creatinine levels to adjust for dilution of samples, often seen during pregnancy as women prepare for pelvic imaging, may improve accuracy in nutrition assessment but no accepted cut-off levels exist [24].

Given these limitations, thyroglobulin (Tg), which is an essential substrate for thyroid hormone synthesis has been considered to be a possible alternative measure of a population iodine nutrition status [25]. In iodine deficient states an increased amount of Tg is released into the circulation [25–29]. It also has been shown to be a good indicator of iodine repletion following iodine intervention allowing monitoring of public health programmes [28, 29].

However, Tg antibodies interfere with the detection of Tg in current assays, causing falsely low Tg measurement and potentially resulting in underestimation of the prevalence of iodine deficiency. Also, current assays lack standardisation with large inter-assay variability and poor reproducibility [30]. Median Tg levels >10 µg/L for iodine sufficiency at a population level were initially reported by the WHO but later removed due to lack of evidence [24, 31, 32]. A recent survey among children established a median of <13 μg/L and/or <3% of Tg samples >40 μg/L as indicative of adequate iodine status [33, 34] More recently, a study among pregnant women with sufficient iodine status as determined by UIC, reported a cut-off median Tg level of ≤10 mg/L and/or <3% of values >44 mg/L as indicative of iodine sufficiency [35]. Increased Tg levels are seen in hyperthyroidism and thyroiditis due to increase activity within the gland [28]. In the early stages of pregnancy, a mild gestational hyperthyroidism is often seen reflecting β-human chorionic gonadotrophin (HCG) stimulation of the thyroid stimulating hormone (TSH) receptor leading to increases in Tg release [35] which may overestimate the prevalence of iodine deficiency during pregnancy. Although this effect generally resolves after the first trimester, its impact, and the ideal timing of Tg measurement among pregnant women is yet to be determined.

This study aimed to recruit pregnant women in NI to monitor their Tg and UIC during pregnancy to establish any possible correlations.

## Materials and methods

The survey was carried out in the Royal Jubilee Maternity Hospital Belfast. Participants were recruited at their booking visit where informed consent was obtained and followed up at scheduled appointments. Exclusion criteria included those under 18 years of age and with known thyroid disease taking thyroid medications. The study received ethical approval from the Office for Research Ethics Committees Northern Ireland (reference 14/NI/0047) and trust governance (13171KM-AS).

Demographic information was collected, and anthropometric measurements recorded. Samples were taken at <14 weeks (1st trimester), ≥18 to ≤22 weeks (2nd trimester) and ≥28 to ≤32 weeks gestation (3rd trimester).

A social deprivation score was collected based on postcode and NI national statistics information. The season, winter (November–April) or summer (May–October), at recruitment was recorded to explore any possible seasonal variation.

A random non fasting spot urine sample was collected at each visit taking care to avoid contamination of samples with the use of urine dipsticks. Samples were frozen at −80° until analysis. UIC was measured using a multiplate persulphate digestion method followed by Sandel-Kolthoff colorimetry as previously described [5]. Urinary creatinine was measured using an established assay and standard operating procedure within our institution. Creatinine concentration was determined using an ILAB 600 chemistry analyser (Werfen, UK) using the Jaffe rate method. This allowed results to be expressed in terms of iodine creatinine ratio (ICR).

A serum sample was taken in each trimester for measurement of Tg and Tg antibody (TgAb), in the Regional Laboratory for Endocrinology at the Royal Victoria Hospital Belfast. Both Tg and TgAb were measured using the Electrochemiluminescence immunoassay (ECLIA) on Elecsys and Cobas e immunoassay analysers (Cobas, Elecsys 2010, Modular analytics E170, Roche Diagnostics GmbH, Sandhofer Strasse 116, D-68305 Mannheim). This method has been standardised against Certified Reference Material (CRM) 457 of the Community Bureau of Reference (BCR) of the European Union. The laboratory reference range for Tg is 0.04–500 ng/ml with values below this range reported as <0.0 4 ng/ml and values above as >500 ng/ml.

The reference range for TgAb was 10.0–4000 IU/ml with values below this range reported at <10 IU/ml and those above as >4000 IU/ml.

Statistical analyses were performed using the Statistical Package for the Social Sciences software (version 23.0; SPSS, Inc. USA) and significance set at *p* < 0.05. Median and interquartile range were used to present non-normally distributed data. Tg, UIC and iodine creatinine ratio (ICR) were not normally distributed, and log transformed for statistical analysis. To compare means between groups an independent *t* test and one-way ANOVA were used. Correlation between variables was assessed using Pearson’s correlation coefficient. Paired samples were compared using a paired *t* test.

## Results

From July 2014 to March 2015 a total of 241 women were recruited to the study and provided an initial urine and serum sample. Two urine samples were excluded from analysis as they had excessive iodine levels, >500 µg/L, most likely following contamination with a urinary dipstick. One hundred and thirty-four urine samples were collected at the 20-week ultrasound scan visit and 158 samples early in the third trimester, a dropout rate of 35% in the third trimester. Corresponding FFQ returns were 240, 130 and 150 at each visit respectively, a completion rate of 62.5% in the third trimester.

Baseline characteristics are outlined in Table 1. The median UIC at the booking visit was 73 μg/L (IQR 38–119 μg/L), on which basis the group was classified as iodine deficient with levels below the WHO cut-off of 150 μg/L [24]. Trimester specific median UICs were 73 µg/L, 94 µg/L and 117 µg/L as previously reported [19]. The corresponding median ICR at each trimester were 116 μg/g, 147 μg/g and 150 μg/g creatinine respectively.

**Table 1:** Patient characteristics at recruitment.

Baseline characteristics (*n*)	
Age in years (241)	Mean 30.3 years (SD 5.4)
Body Mass Index kg/m^2^ (224) Height cm (224) Weight kg (224)	Mean 26.2 kg/m^2^ (SD 4.9) Mean 163.7 cm (SD 6.5) Mean 70.1 kg (SD 13.9)
Occupation Higher (92) Intermediate (53) Lower/UE/ill-defined (77)	41.4% 23.9% 34.7%
Deprivation Scores 1 (Most affluent) (61) 2 (48) 3 (31) 4 (18) 5 (Most deprived) (83)	25% 20% 13% 8% 34%
Smoking Never (214) Current (25) Ex-smoker (2)	88.8% 10.4% 0.8%
Ethnicity White Caucasian (238) Black African (2) Indian (1)	98.8% 0.8% 0.4%
Previous pregnancy 0 (114) 1–2 (106) >2 (21)	47.3% 44.0% 8.7%
Previous miscarriage Yes (74) No (167)	30.7% 69.3%
Planned pregnancy Yes (189) No (52)	78.4% 21.6%
Diet Normal (225) Vegetarian (6) Gluten free (6) Other	93.4% 2.5% 2.5% 1.6%
Family history of thyroid disease Yes (51) No (170)	21.2% 70.5%
Iodine-containing multivitamin Yes (127) No (105)	52.7% 43.6%
Season Winter (151) Summer (90)	62.7% 37.3%

### Thyroglobulin (Tg)

After exclusion of TgAb positive participants (*n* = 15), 224 participants had Tg suitable for analysis (Table 2). The median Tg level for all samples was 17 μg/L (*n* = 473, IQR 11.0–31.0) with 12.5% ≥44 µg/L. The median trimester specific Tg levels were 19 µg/L, 16 µg/L and 16 µg/L with corresponding proportion of samples with a Tg level ≥44 µg/L of 14.3%, 9.4% and 12.4%. These values are consistent with iodine deficiency using recently defined cut-off values [36].

**Table 2:** Thyroglobulin measurements during pregnancy by gestational period Tg: thyroglobulin.

Gestation (weeks)	Number of samples	Median Tg (IQR)	%Tg ≥ 44 µg/L
**<14**	203	19.0 (12.0–32.0)	14.3
**≥18** **≤** **22**	117	16.0 (11.0–28.5)	9.4
**≥28** **≤** **32**	113	16.0 (10.0–29.5)	12.4

*IQR* interquartile range.

Paired samples showed that median Tg levels were significantly higher in the first trimester (19 µg/L) compared with both the second (16 µg/L, *n* = 120, *P* = 0.001) and third trimesters (16 µg/L, *n* = 118, *P* = 0.021). There was no significant difference between the second and third trimester Tg levels. Non-smokers (*n* = 214) had significantly lower Tg levels in the first trimester than current or ex-smokers (18 µg/L vs. 27 µg/L), but current or ex-smokers were few.

Reported planning of the pregnancy was associated with a lower Tg level (17.4 µg/L vs. 23.3 µg/L) but did not reach significance (*P* = 0.052). Tg was not associated with occupation, parity, previous miscarriages, diet, presence of ongoing vomiting after first trimester and family history of thyroid disease. There were also no significant associations between Tg and consumption of iodine-containing supplement during the first trimester, and seasonal variation observed in any trimester.

Overall, there were significant correlations with Tg and UIC (*r* = −0.262, *P* < 0.001), ICR (*r* = −0.147, *p* < 0.01) and TSH (*r* = −0.134, *p* < 0.01). During the first trimester, Tg was negatively correlated with UIC (*r* = −0.258, *P* < 0.001), urinary ICR (*r* = −0.277, *P* = 0.002), TSH (*r* = −0.147, *p* < 0.05) and free T4 (*r* = 0.178, *p* < 0.05). In the second trimester the only significant associations were for UIC (*r* = −0.217 *p* < 0.05) and ICR (*r* = −0.280 *p* < 0.01). None of the correlations reached significance in the third trimester.

## Discussion

Our study confirms that this cohort of iodine deficient pregnant women in NI as defined by median UIC and ICR had both a median Tg >10 μg/L and a high number with values ≥44 μg/L across all trimesters.

Median UIC values of ≤150 μg/L were similar to previous UK studies in pregnant women [4], including one UK study by Bath et al. which reported Tg as a measure of iodine deficiency [37]. To our knowledge, studies outside of the UK, in iodine deficient areas, have shown similar thyroglobulin levels across pregnancy [26]. One group noted that women with sufficient iodine intake had stable Tg levels during pregnancy, while women with mild iodine deficiency showed a rise in Tg throughout gestation associated with an increase in thyroid volume [38]. This suggests thyroid stimulation due to the iodine deficient state. Tg levels were significantly higher in women with moderate iodine deficiency when compared to those who were iodine sufficient and correlated with UIC. Table 3 shows a summary of pregnancy studies which measure Tg in iodine deficiency.

**Table 3:** Comparison with similar studies of Tg in pregnancy.

Author (reference)	Year	Country	Number (*n*)	UIC < 150 µg/L (measure of adequacy)	Tg ng/ml 1st T	Tg 2nd T	Tg 3rd T	Tg assay
Costeira et al. [41]	2010	Portugal	118	×	11	11	13	RIA
Brucker-Davis et al. [42]	2012	France	110	×	17	–	–	IRMA
Raverot et al. [26]	2012	France	228	×	17	16	16	IRMA
Andersen et al. [43]	2013	Denmark	140	×	–	–	23	ILMA
Brough et al. [44]	2013	New Zealand	70	×	–	–	16	ICMA
Katko et al. [45]	2014	Hungary	189	×	–	14	–	CLIA
Bath et al. [37]	2015	England	230	✓	21	19	23	CLIA
Stinca et al. [31]	2017	11 non-EU counties^a^	3870	^b^=	17	19	19	EIA
Censi et al. [38]	2019	Italy	38 Placebo	×	8	8	10	CLIA
			52 Iodine	×	8	8	6	CLIA
Current	2021	N. Ireland	241	×	19	16	16	CLIA

*UIC* urinary iodine concentration, *T* trimester, *Tg* thyroglobulin, *EU* European Union, ✓ sufficient, = borderline sufficiency, × insufficient. *RIA* Radioimmunoassay, *IRMA* Immunoradiometric assay, *ILMA* Immunoluminometric assay, *ICMA* Immunochemiluminometric assay, *CLIA* Chemiluminescent immunoassay, *EIA* Enzyme immunoassay.

^a^Morocco, Niger, Philippines, Croatia, Switzerland, Croatia, Thailand, South Africa, China, Nepal, India, Tanzania.

^b^The group studied by Stinca et al. was initially iodine sufficient with a UIC of 152 µg/L however this fell below the threshold for sufficiency in the second trimester [31].

Tg levels were higher in the first trimester, similar to other studies. This may indicate a degree of gestational hyperthyroidism through the action of β-HCG although this has mainly been shown in iodine deficient studies and may represent a compensatory mechanism [35]. However, levels did not significantly change from second to third trimesters which may indicate that these compensatory mechanisms are most marked when adequate thyroid hormone function is most critical for foetal development.

Both UIC and ICR increased through pregnancy. This may be due to the likelihood of dilute urine samples in early pregnancy and increased glomerular filtration during the latter stages. Tg levels showed less variation beyond the first trimester.

In our study we found a significant relationship between Tg and UIC and ICR. Studies in larger groups have found a U-shaped relationship with high Tg levels for both low and high UIC [32, 39]. This suggests there is a window of optimal iodine intake. Some have suggested that commencing iodine supplementation during pregnancy may have an adverse effect on thyroid function [40]. This highlights the importance of eliminating iodine deficiency at a population level, particularly given the re-emergence of iodine deficiency in schoolgirls in the UK.

Significant negative associations were seen with smoking in the first trimester. Smoking is known to inhibit iodine uptake, although the small number of ex or current smokers in this study limited any definite conclusions [17]. Unlike urinary iodine, neither iodine supplementation nor season had any association with Tg. This may be expected though as Tg is a chronic marker of iodine nutrition and unlikely to be influenced significantly by season and recent iodine supplementation.

Correlations were strongest in the second trimester between ICR and Tg. As ICR has been demonstrated to be a better marker of iodine nutrition during pregnancy [4] and Tg improved after the first trimester but levelled off, we feel that the ideal time for Tg measurement may be mid second trimester although further studies are needed to evaluate this.

Documented concerns regarding the reliability of results due to TgAb interference are considered in this study, but the prevalence of antibodies was low at 5.9% with a decrease in the second (3.9%) and third trimesters (2.3%), similar to other studies [26, 30]. Whilst antibodies may interfere with the assessment of individual iodine status, they are unlikely to interfere with assessment at a population level [31]. Higher TgAbs have been found in countries with iodine deficiency. Our prevalence of TgAb positive women is lower than expected but mild deficiency is less likely to be associated with such high levels.

The strengths of the study include recruitment of participants throughout the entire year thus reducing seasonal variation which has been seen with other measures. Participants were asked to provide serial samples allowing us to assess trends in individuals through their pregnancy. Although this was not an intervention study, many participants were taking an iodine-containing supplement (*n* = 127). Although differences in Tg levels were observed, overall median levels even in those taking such supplements were above the recommended cut-off levels (19.1 µg/L vs. 18.3 µg/L with *p* = 0.730).

The main limitation of this study was a relatively high dropout rate (38%).

## Conclusion

Our results would suggest that Tg is a good measure of iodine status and thyroid function during pregnancy and, potentially, the best measure during the second trimester. Further work to validate the cut-off values for iodine sufficiency in pregnancy are required. UIC has not been validated during pregnancy and, given the changes known to occur that alter iodine handling the use of current cut-off values, which were extrapolated from studies in children, may lead to incorrect interpretation of iodine status during pregnancy. Tg may offer a complementary measure of longer-term iodine status along with its effect on thyroid function.

## Data Availability

Data generated during this study is included in this published article. Additional data can be requested from the corresponding author.
